# Cognitive neuropsychiatric analysis of an additional large Capgras delusion case series

**DOI:** 10.1080/13546805.2019.1584098

**Published:** 2019-02-22

**Authors:** Emily A. Currell, Nomi Werbeloff, Joseph. F. Hayes, Vaughan Bell

**Affiliations:** a Division of Psychiatry, University College London (UCL), London, UK; b Institute of Psychiatry, Psychology and Neuroscience, King’s College London, London, UK; c Camden and Islington NHS Foundation Trust, London, UK; d South London and Maudsley NHS Foundation Trust, London, UK

**Keywords:** Delusional misidentification, psychosis, schizophrenia, neuropsychiatry, forensic

## Abstract

**Introduction:**

Although important to cognitive neuropsychiatry and theories of delusions, Capgras delusion has largely been reported in single case studies. Bell et al. [2017. Uncovering Capgras delusion using a large scale medical records database. *British Journal of Psychiatry Open*, *3*(4), 179–185] previously deployed computational and clinical case identification on a large-scale medical records database to report a case series of 84 individuals with Capgras delusion. We replicated this approach on a new database from a different mental health service provider while additionally examining instances of violence, given previous claims that Capgras is a forensic risk.

**Methods:**

We identified 34 additional cases of Capgras. Delusion phenomenology, clinical characteristics, and presence of lesions detected by neuroimaging were extracted.

**Results:**

Although most cases involved misidentification of family members or partners, a notable minority (20.6%) included the misidentification of others. Capgras typically did not present as a monothematic delusion. Few cases had identifiable lesions with no evidence of right-hemisphere bias. There was no evidence of physical violence associated with Capgras.

**Conclusions:**

Findings closely replicate Bell et al. (2017). The majority of Capgras delusion phenomenology conforms to the “dual route” model although a significant minority of cases cannot be explained by this framework.

## Introduction

The Capgras delusion is the delusional belief that another person, often a partner or family member, has been replaced by an identical or near-identical looking impostor (Ellis & Young, 1990). It has typically been reported in the context of both neurological damage and psychiatric disorder (Edelstyn & Oyebode, 1999) and is considered rare—generally being reported as single case studies in the medical literature (Christodoulou, Margariti, Kontaxakis, & Christodoulou, 2009).

Although an uncommon psychiatric disorder, Capgras delusion has been central to the development of theories of delusions and has been foundational to the field of cognitive neuropsychiatry (Bell, Halligan, & Ellis, 2006; Halligan & David, 2001). Perhaps the most influential model of Capgras delusion builds on the dual route model of face recognition (e.g. Bruce & Young, 1986; Ellis & Lewis, 2001) that argues for two routes to face recognition—one conscious and one implicit-affective. The “dual route” model of face recognition was based on data showing that patients with acquired prosopagnosia were unable to effectively distinguish familiar from non-familiar faces in face recognition tests but nevertheless demonstrated a reliable autonomic skin conductance response to familiar faces (Bauer, 1984) suggesting both conscious and unconscious routes to recognition. Ellis and colleagues first predicted (Ellis & Young, 1990) and later confirmed (Ellis, Quayle, & Young, 1999; Ellis, Young, Quayle, & De Pauw, 1997) that Capgras patients would show the reverse dissociation between measures, indicating an intact conscious face recognition route but an impaired implicit-affective route.

Ellis and Young (1990, 1997) argued that this pattern of impairment could provide the initial experience of “known people feeling unfamiliar” that formed the basis of the delusional belief that familiar people had been replaced. The fact that this belief was not rejected on its unlikely basis suggested that an additional impairment to a “second factor”—the ability to reason effectively about anomalous experiences—was also needed for a delusional belief to form (Coltheart, 2007). Although some theories of Capgras have taken an entirely different tack—for example, Wilkinson’s (2016) mental files approach and Margariti and Kontaxakis (2006) model of Capgras as a disorder of the sense of uniqueness—most current explanations build upon Ellis and Young’s dual route model of Capgras delusion (e.g. Coltheart, Menzies, & Sutton, 2010; Pacherie, 2009; Young, 2008).

Importantly, Ellis and Young’s model of Capgras delusion has a specific explanatory scope. Because it explains how familiar people are recognised with a concurrent sense of emotional unfamiliarity, it can only explain the misidentification of known people and cannot explain where previously unfamiliar people are believed to have been replaced. However, exactly this presentation was reported in Capgras and Reboul-Lachaux’s (1994) original case study where *Madame M.* believed her family had been replaced by impostors but also believed residents of Paris and the “whole world” had been replaced. It has also been reported as a minority presentation of Capgras delusion in the literature since (see reviews in Berson, 1983; Pandis *et al*., 2019) although the extent to which Capgras delusion solely presents as non-familiar person misidentification, or is accompanied by non-familiar person misidentification, has been difficult to assess systematically.

Capgras has also been associated with neurological disorder to the point where several authors have argued that all individuals with Capgras should be investigated for organic pathology (Christodoulou, 1977; Maharajh & Lutchman, 1988). Evidence from neuroimaging has suggested a link between Capgras and right hemisphere abnormalities, especially of the frontotemporal regions, as revealed by magnetic resonance imaging (MRI) (Christodoulou, 1991; Dietl, Herr, Brunner, & Friess, 2003; Edelstyn & Oyebode, 1999; Forstl, Almeida, Owen, Burns, & Howard, 1991). In a literature review of 26 patients with Capgras who had organic factors implicated (Feinberg & Shapiro, 1989), the majority had bilateral lesions although for those with unilateral lesions, right hemisphere lesions were much more likely.

However, because Capgras delusion has most commonly been reported as single case studies, it has been difficult to make systematic inferences about such clinical characteristics and pathophysiology. Nevertheless, two recent studies have attempted to analyse large case series. Salvatore et al. (2014) reported 73 Capgras cases out of 517 episodes of first-episode psychosis. However, they relied on single raters identifying cases based on the definition “a delusional belief in the existence of virtually identical ‘doubles’ of persons significant to a patient or the patient him- or herself” that potentially encompasses several delusional misidentification syndromes and not solely Capgras—which may explain their surprisingly high reported level of prevalence of 14.1%.

Taking advantage of the availability of anonymised data from electronic health records for psychiatric research, Bell et al. (2017) used structured criteria, inter-rater classification, and computational data extraction to identify cases in the electronic records of over 250,000 people from a regional mental health service in South London. Although this study could not estimate prevalence, it used a high sensitivity strategy to identify a large series of 84 cases. Bell et al. (2017) reported that most cases involved misidentified family members and close partners but others were the subject of misidentification in 25% of cases, contrary to the dual route theory of Capgras. Furthermore, Capgras was accompanied by other delusions in the majority of cases and so was rarely an example of a “monothematic” delusion, as has previously been suggested (Coltheart, 2013). Examination of reported neuroimaging results provided no evidence of predominantly right hemisphere damage.

In this study, we aimed to replicate Bell et al. (2017) using a near-identical system for conducting research on anonymised medical records but focusing on a distinct population in North London. In addition to the extracting the same information as the original Bell et al. study to further test cognitive neuropsychiatric theories of Capgras delusion, we also collected information on whether there was evidence of verbal or physical aggression against the subject of delusional replacement. Capgras has been described as “frequently” involving violence towards the perceived impostor and has been recommended as a risk marker in psychiatric assessments (Bourget & Whitehurst, 2004; Carabellese, Rocca, Candelli, & Catanesi, 2014; de Pauw & Szulecka, 1988; Horn et al., 2018; Silva, Harry, Leong, & Weinstock, 1996) although, until now, conclusions have been based on published case studies that may be subject to significant reporting bias.

## Methods

The study used a version of the Clinical Record Interactive Search (CRIS), an anonymised electronic health record database of patients that covered medical records from patients presenting to the Camden and Islington NHS Foundation Trust. This NHS Trust is the public provider of secondary and tertiary mental health care in North London covering two inner-city London borough of Camden and Islington with a catchment population of approximately 470,000 individuals. Anonymised electronic records are available to approved researchers through the CRIS system that holds records for over 120,000 people. Full details of the patient cohort covered by this CRIS system, including demographics and clinical features, are reported in Werbeloff et al. (2018). This CRIS system is covered by ethical approval granted by the National Research Ethics Service Committee East of England—Cambridge Central (reference 14/EE/0177).

### Case identification

Following the approach of Bell et al. (2017) we conducted a preliminary keyword search using the word “Capgras” to check whether it had sufficient scope for identifying potential cases. Informal inspection indicated that this only retrieved a small number of cases and so we additionally included “misidentification” as an independent retrieval keyword to identify records for further manual case identification. Retrieved records were then rated for the presence of the Capgras delusion by two independent raters using the structured classification in Table 1 modified from Bell et al. (2017) to include the keyword misidentification. The two independent raters were postgraduate students at the University College London Division of Psychiatry, trained by author VB, who was a rater on the earlier study.

**Table 1.** Categories and definitions for case note classification used by independent raters.

**Strongly** Capgras or misidentification delusion is mentioned as a present delusion plus evidence of present or recent relevant delusional misidentification of people is described (mention of people being replaced, or impostors, or lookalikes, or identical looking people, or clones, or robots etc)
**Possibly** Capgras or misidentification delusion is mentioned as a present delusion but no additional description of delusion content is given, or delusional nature is questioned, or the description is clearly not person misidentification.
**Not Present** Capgras or misidentification delusions are excluded, or mentioned erroneously, or conflicts with the description of the delusion (clearly not misidentification)
**Only Past** Capgras or misidentification delusions are only mentioned as previously present with no evidence of current misidentification, or is described as fully resolved.

We therefore identified a case of Capgras based on two criteria: (i) the clinician describes the patient as having a misidentification delusion or a Capgras delusion, and (ii) the delusional misidentification is described as involving someone being replaced, or impostors, or lookalikes, or identical looking people, or clones, or robots etc. Following Bell et al. (2017), only cases meeting the criteria for “strongly” indicating the presence of Capgras delusion according to the rating system were included. The date of the record from which raters first identified strong evidence of Capgras (referred to as date of “Case ID”) for each case was noted.

**Figure 1.**
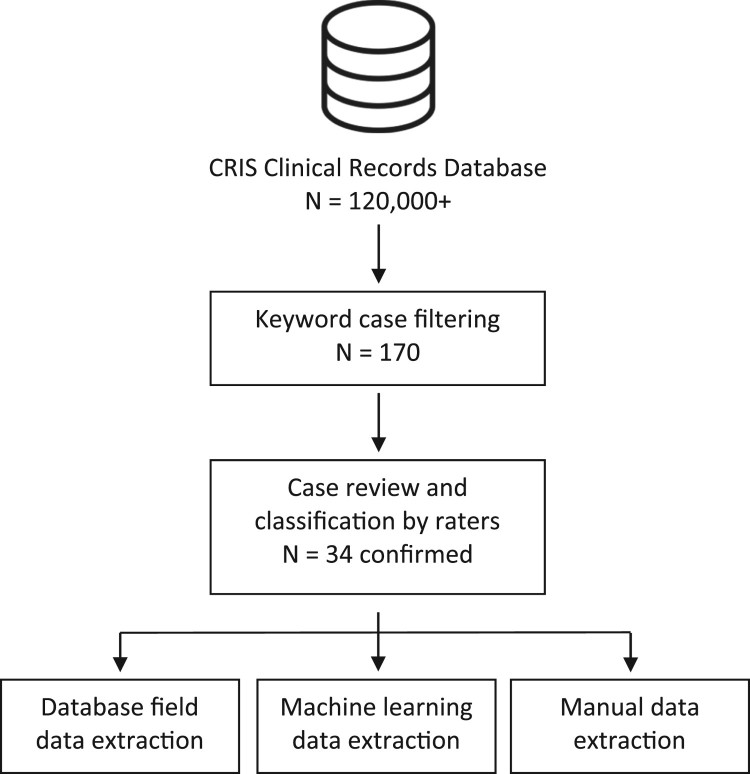
Capgras case data extraction procedure.

The level of independent agreement between raters was assessed with Cohen’s kappa and disagreements after independent rating were resolved through discussion. The data extraction procedure is illustrated in Figure 1.

### Data extraction

For the identified cases, demographic information and primary diagnosis were extracted from database fields. Age at the time of presentation with the Capgras delusion was calculated as time elapsed from date of birth to Case ID. Clinical information was extracted from structured and unstructured fields. Clinical information extracted from structured fields included psychiatric diagnosis at time of Case ID and diagnosis 6 months from Capgras presentation. Data for neuroimaging assessment results were extracted from reports arising from the assessments. Unlike in the original Bell et al. (2017) study, MRI scan reports were not available due to a database reset during the final stages of data extraction so neuroimaging results were limited to computed tomography (CT) and electroencephalography (EEG) results. Delusion phenomenology, working diagnosis, additional delusions, and the presence of formal thought disorder or hallucinations were collaboratively extracted by the two raters from written records.

Mini Mental State Examination (MMSE) scores were extracted from unstructured text using the General Architecture for Text Engineering (GATE) natural language processing system—a machine learning framework that performs text-analysis of human language. The MMSE extraction application has been tested in the C&I NHS Trust health records database and has high positive predictive value (i.e. precision) of 98% and sensitivity (i.e. recall) of 94% was reported, although recall accuracy of dates was substantially lower at 67% overall (Aworinde, Werbeloff, Lewis, Livingston, & Somerland, 2018).

GATE was used also to extract antipsychotic medication history. The antipsychotic prescribing extraction application has not been tested for sensitivity and specificity in C&I NHS Trust health-records database. However, the same GATE software has been tested in the SLaM Case Register; Kadra et al. (2015) reports high precision (ranging from 0.94 to –0.97) but variable recall (as low as 0.57 for haloperidol and as high as 0.92 for clozapine). Poor recall for antipsychotic extraction may result in underestimation of antipsychotic prescribing.

## Results

### Inter-rater reliability of ratings

For case classification, the Cohen’s kappa for independent agreement between raters was 0.73 indicating an acceptable level of agreement between raters. 34 cases were identified as having strong evidence for presence of the Capgras delusion and were included for further analysis.

### Demographics

Of the 34 patients, the majority were female (64.7%, *N *= 22). The mean age at time of case ID was 50.0 years of age (*SD *= 20.2, range 18–88). Ethnicity of the cases was classified as White British, White European or White Other (*N* = 18), Black British, Black British Somali, Black British Carribean, Black British Nigerian or Black Pakistani (*N* = 11), Asian or Asian British (*N* = 3), “Other” (*N* = 1) with one case with missing ethnicity data. For country of origin, 11 cases had no country of origin listed, 10 were listed as African or Caribbean in origin, 8 as British, 3 as European, 1 as American (USA) and 1 as the United Arab Emirates.

### Diagnosis

Existing primary diagnosis, working diagnosis and recoded diagnosis 6 months after Case ID are reported in Table 2. Notably, cases are most likely to transition to a diagnosis of schizophrenia after 6 months.

**Table 2.** Diagnoses of identified Capgras cases at time of Case ID and six months after.

	Existing diagnosis on presentation		Working diagnosis on presentation		Formal diagnosis 6 months after presentation	
Diagnosis	N	%	N	%	N	%
None recorded	10	29.4%	4	11.8%	0	0.0%
Schizophrenia, schizotypal, and delusional disorders (F20-29)	11	32.4%	18	52.9%	23	67.6%
Organic (F00-09)	5	14.7%	6	17.6%	5	14.7%
Mood disorders (F30-39)	7	20.6%	5	14.7%	5	14.7%
Mental and behavioural disorders due to substance use (F10-19)	1	2.9%	1	2.9%	1	2.9%

### Antipsychotic prescribing

31 out of the 34 cases (91.2%) had a recorded antipsychotic prescription history. The most commonly prescribed antipsychotic medications were risperidone (21 cases; 67.7%), olanzapine (19 cases; 61.3%), and haloperidol (14 cases; 45.2%). Also prescribed were quetiapine (9 cases; 29.0%), clozapine (9 cases; 29.0%), flupenthixol (7 cases; 22.6%), zuclopenthixol (6 cases; 19.4%), aripiprazole (5 cases; 16.1%), amisulpride (5 cases; 16.1%), fluphenazine (2 cases; 6.5%), chlorpromazine (2 cases; 6.5%), sulpiride (2 cases; 6.5%), paliperidone (1 case; 3.2%), and pipotiazine (1 cases; 3.2%).

### Symptom phenomenology

Symptom phenomenology was extracted and categorised collaboratively by two raters from the record text. Categories were not exclusive and therefore category totals sum to more than the total number of cases in some instances.

The reported subject of the delusional replacement was a family member or close partner in 30 cases (88.2%) out of 34 cases. Non-family or non-partner misidentification was reported in 7 cases (20.6%). These included friends in 2 cases (5.9%), neighbours in 2 cases (5.9%), health care providers in 2 cases (5.9%), and “police” and “the population of Great Britain” in 1 case each (each 2.9% of cases). 4 cases (11.8%) had non-family and non-partner misidentification only and 3 (8.8%) had a combination of both. 11 cases (32.4%) were reported as involving the replacement of more than one person.

The identity of the “replacer” was described in terms of a specific agent or agents in 5 cases (e.g. “someone called [female name]”, “police officers”, “neighbours”, “friends”). A supernatural agent featured as a replacer in one case (“jinn”).

Patient-reported justification for the replacement was most often not reported (*N* = 21, 61.8%). In those that did report a justification, the most common was a perceived physical difference in the impostor in 6 cases (17.7%), a sense of unfamiliarity in 2 cases (5.9%), strange interactions / the person not their usual self in 2 cases (5.9%), a perceived alteration of emotional responsivity by the impostor in 2 cases (5.9%,), and a perceived slight difference in personality or character in 1 case (2.9%).

In 25 cases (73.5%) delusional beliefs in addition to the Capgras delusion were reported whereas the remaining 9 cases (26.5%) had Capgras as the only reported delusion.

In 15 cases (44.1%) recent or current hallucinations were reported, in 7 cases (20.6%) hallucinations were assessed and excluded, and hallucinations were not mentioned in 12 cases (35.3%). Of the cases where hallucinations were reported, 11 cases (73.3%) reported auditory hallucinations. Over a third of patients had evidence of formal thought disorder (38.2%, *n *= 13), which was described as tangentiality in 6 cases, flight of ideas in 3 cases, loosening of associations in 2 cases, derailment in 2 cases, circumstantiality in 2 cases, and pressured speech in 1 case.

### Association with risk

Perceived malicious intent of the “impostor” was reported in 14 cases (41.2%). Verbal abuse or verbal hostility towards the person identified as the “impostor” was reported in 6 cases (17.7%). No cases had reported physical aggression or violence.

### Cognition

Mini Mental State Examination (MMSE) was reported in 10 of the 34 cases. Mean MMSE score for Capgras cases was 22.4 (*SD *= 5.1, range 11.6–30), indicative of mild cognitive impairment (MCI). 40% of patients had a “normal” MMSE score of 24 points or above (*N *= 4). 40% had a score indicative of mild cognitive impairment, which is in the 18–23 range (*N *= 4), and 20% had a score indicative of moderate cognitive impairment (10–17 range) (*N *= 2).

### Neuroimaging assessment results

MRI results were not retrievable due to the medical records database being rebuilt and re-anonymised during the research project. CT head scans were available for 7 of the 34 cases, 5 of which were deemed abnormal. CT results for the abnormal scales are reported in Table 3. Of the abnormal CT scans, only one presented with focal neuropathology for which lateralisation was not reported. All other presented with minor abnormalities likely reflecting non-specific changes. There was no evidence for the predominance of right lateralised neuropathology. No EEG results were found for the 34 cases of Capgras.

**Table 3.** CT scan results with reported abnormalities from Capgras delusion cases.

Case No.	Age	Days from Case ID	Dx at Case ID	Lateralisation	Results
1	85	0	Delusional disorder	Unknown	Localised area of obliteration of surface marking top of superior frontal gyrus (extra axial calcified lesion, possibly secondary to meningioma)
2	74	−80	Alzheimer’s	Bilateral	Minor diffuse prominence of CSF space
3	87	1	Early dementia	Right	Generalised involutional change and multiple confluent low densities seen in the deep, right ventricle and subcortical white matter in keeping with an established small vessel ischaemia
4	66	−857	Delusional disorder	Bilateral	Moderate distension involutional change with central ventricular dilation and widening of the peripheral CSF spaces. Minimal low attenuation change in the deep white matter.
5	88	−271	Alzheimer’s	Bilateral	Moderate age appropriate cerebral atrophy with some periventricular hypodensity suggestive of small vessel disease. Frontal and temporal atrophy. Symmetrical widening of ventricles and cortical sulci (age-related involution), slightly more prominent in frontal lobes.

## Discussion

We identified 34 cases of Capgras delusion in a large health-care records database from a major London provider of secondary and tertiary public mental health services. We found comparable results to Bell et al. (2017), namely, that a significant minority of cases do not conform to the “dual route” model of Capgras delusion, that Capgras is typically not a “monothematic” delusion, and that right hemisphere damage is not predominant in cases that present to psychiatric services.

In this study, the most commonly misidentified subject was a family member or close partner. However, in a notable minority of cases, the misidentified subject was a non-family or non-partner individual. This was reported in 7 out of 34 cases (20.6%) and there were 4 cases (11.8%) that had exclusively non-family or non-partner misidentification. This echoes findings from Bell et al. (2017) where 21 out of 84 (25%) involved non-family or non-partner misidentification and 12 (14.3%) involved non-family or non-partner misidentification exclusively.

With regard to the Ellis and Young model of Capgras delusion, there are clearly cases of misidentification of strangers that do not conform to the phenomena within the model’s scope of explanation. This is in line with data reported in Bell et al. (2017) and in line with previously reported cases in the literature (Berson, 1983; Pandis et al., forthcoming). We previously noted that this suggests two alternatives: (i) that “familiar-person Capgras” and “non-familiar person Capgras” are distinct syndromes that might need neuropsychologically distinct explanations; or (ii) that the Ellis and Young model of Capgras delusion needs modifying to include the possibility of misidentifying non-familiar people. We also note a third, that the Ellis and Young explanation may simply be wrong, although we find this least likely given the independently replicated evidence for its central empirical prediction of impaired autonomic response to familiar faces (Brighetti, Bonifacci, Borlimi, & Ottaviani, 2007; Hirstein & Ramachandran, 1997).

It is worth noting that this debate can also be reframed in terms of the definition of Capgras—in that we could accept that “Capgras delusion” refers solely to familiar-person Capgras and delusions regarding impostors replacing non-familiar persons could be given an alternative moniker. This would have the advantage of defining Capgras in line with its use in the most recent literature (e.g. Christodoulou et al., 2009; Davies, Coltheart, Langdon, & Breen, 2001; Edelstyn & Oyebode, 1999) but would run counter to the canonical case of Capgras and Reboul-Lachaux (1994), previous definitions of Capgras (de Pauw, 1994), and its use in prior reported cases (Berson, 1983). We note that this is a classic “lumping or splitting” debate in classification but leaves the scientific arguments unchanged—that is, which explanations best fit which phenomena.

These possibilities do lead to a clear empirical prediction however: that if the scope of the Ellis and Young model is specific to familiar persons, patients who *solely* present with non-familiar person Capgras delusion will not show the lack of autonomic response to familiar faces. This may be difficult to test in practice, however, as this group of patients appear to be an uncommon subset of a rare syndrome.

Perhaps it is also worth stating an implicit but important conclusion from the results reported both here and in Bell et al. (2017)—that the majority of patients not only conform to the definition needed for the Ellis and Young model and also to the narrower description of Capgras where the majority of subjects of replacement are not only familiar people but are family members or partners. Those most familiar (family, partners) are most likely to be misidentified, followed by casual acquaintances (neighbours, clinical staff) and finally, least commonly of all, undifferentiated strangers (everyone, “the police” etc). Indeed, it seems the chance of being misidentified in Capgras delusion is an inverse function of familiarity, or potentially, an inverse function of the strength or richness of the individuated mental representation for the people concerned. This may be a novel avenue for future research.

We also found that the majority of cases of Capgras delusion presented with additional delusional beliefs aside from Capgras, indicating that Capgras is not best classified as a monothematic delusion (Davies et al., 2001). Here, we suggest that rather than defining Capgras delusion as monothematic, cases are simply described as presenting with monothematic Capgras or Capgras as part of a polythematic delusional system.

In contrast to previous reports that have associated Capgras delusion with dangerousness (e.g. Carabellese et al., 2014; de Pauw & Szulecka), we found no cases of Capgras delusion where violence was reported and only 6 out of 34 that reported verbal hostility towards the subject of delusional replacement. Due to the focus on risk management in secondary and tertiary mental health services, we assume risk-related behaviour would be among the better-documented clinical features.

This raises the question to what extent Capgras has been over-associated with violence in the existing literature. Notably, claims for this association in the literature are remarkably strong. For example, numerous authors (Bourget & Whitehurst, 2004; Carabellese et al., 2014; de Pauw & Szulecka, 1988; Horn et al., 2018; Silva et al., 1996) claim that Capgras may be a specific risk factor for violence, and even murder, of the misidentified person. These claims are particularly notable given they are largely based on a literature formed mainly of single cases or small case series that may be subject to significant reporting bias. The extent to which Capgras delusion may be a risk factor for violence may be hard to determine given its potential rarity—which has traditionally precluded epidemiological studies. However, approaches using case identification on large medical records databases, potentially across sites, may be able to test this association even for rare cases in future studies.

With regard to neuroimaging findings, a more limited range of clinical assessments was available compared to Bell et al. (2017), although the main conclusion was broadly similar. Namely, that there was no evidence of right-hemisphere damage being predominant in cases of Capgras delusion presenting to mental health services. Some researchers have previously argued that Capgras may be fundamentally an “organic” syndrome (Christodoulou, 1977). However, our results seem to support the distinction made by researchers such as Malloy, Cimino, and Westlake (1992) and Darby and Prasad (2016) between lesion- and non-lesion-related Capgras.

We also found a wide range of variability in ethnic background, age and MMSE score. Notably we found a similar gender ratio of approximately 2:1 female to male as the original Bell et al. (2017) study.

In terms of limitations, the method we used in this and our previous study is based on information collected as part of routine clinical practice. Although core clinical information stored in specific database fields is likely to be accurate, notes stored as free text will contain information that depends on clinician preference and accuracy. Additionally, the clinician’s interpretations may not be representative of the patient’s experience or may have been influenced by service priorities or theoretical perspectives dominant at the time. The results of neuroimaging were extracted from radiology reports as the original scans are not available to researchers. In addition, due to technical issues we were not able to extract results from MRI scans, meaning there may have been lesions that may have been detected on MRI that would not be adequately detected by CT scans. Unlike the previous study, which used experienced clinicians to rate cases, this study used trained postgraduate students to identify cases. However, we note here that the inter-rater reliability on both occasions was similar, indicating a similar level of classification performance. Importantly, we were unable to estimate prevalence of the Capgras delusion. We utilised a high sensitivity search strategy to identify cases of Capgras but it is highly likely that this approach missed many true cases.

When combined with the findings of Bell et al. (2017) we have identified a total of 118 cases of Capgras delusion across two distinct mental health services in two geographically close and ethnically diverse areas of London. Both samples show similar results in terms of lack of monthematicity and right-sided lesion prevalence, and both studies report cases of non-familiar person misidentification, alone, and in combination with, familiar person misidentification, that are not easily accounted for by the dual route model of Capgras. In addition, in this new study, we did not find evidence for a consistent association with dangerousness as has been claimed in previous reports.
